# Impact of Haemoadsorption Therapy on Short Term Mortality and Vasopressor Dependency in Severe Septic Shock with Acute Kidney Injury: A Retrospective Cohort Study

**DOI:** 10.3390/antibiotics13121233

**Published:** 2024-12-22

**Authors:** Danny Epstein, Karawan Badarni, Yaron Bar-Lavie

**Affiliations:** 1 Critical Care Division, Rambam Health Care Campus, Haifa 3109601, Israely_barlavie@rambam.health.gov.il (Y.B.-L.); 2 Ruth and Bruce Rappaport Faculty of Medicine, Technion, Haifa 3525433, Israel

**Keywords:** septic shock, cytokine removal, acute kidney injury, continuous renal replacement therapy, haemoadsorption, Cytosorb, Oxiris

## Abstract

**Background/Objectives**: Sepsis, a life-threatening organ dysfunction caused by a dysregulated host response to infection, remains a major challenge in ICUs. This study evaluated whether combining haemoadsorption therapy with continuous renal replacement therapy (CRRT) reduces ICU and short-term mortality in patients with severe septic shock and acute kidney injury (AKI) requiring CRRT. **Methods**: A single-centre retrospective cohort study was conducted at Rambam Health Care Campus, Haifa, Israel, from January 2018 to February 2024. Data were collected from ICU patients with severe septic shock and AKI requiring CRRT. Patients were divided into two groups: those receiving haemoadsorption therapy with CRRT and those receiving CRRT alone. Primary and secondary endpoints included ICU, 30 and 60-day mortality, vasopressor dependency index (VDI), and lactate levels. **Results**: Out of 545 patients with septic shock, 133 developed AKI requiring CRRT, and 76 met the inclusion criteria. The haemoadsorption group (*n* = 47) showed significant reductions in blood lactate levels and VDI after 24 h compared to the CRRT alone group (*n* = 29). ICU mortality was significantly lower in the haemoadsorption group (34.0% vs. 65.5%, *p* = 0.008), as was 30 and 60-day mortality (34.0% vs. 62.1%, *p* = 0.02, and 48.9% vs. 75.9%, *p* = 0.002). Multivariate analysis confirmed haemoadsorption therapy as independently associated with lower ICU and 30-day but not 60-day mortality. **Conclusions**: Haemoadsorption therapy combined with CRRT in patients with severe septic shock and AKI requiring CRRT is associated with improved lactate clearance, reduced vasopressor requirements, and lower ICU and 30-day mortality. Further high-quality randomized controlled trials are needed to confirm these findings.

## 1. Introduction

Sepsis, defined as a life-threatening organ dysfunction caused by a dysregulated host response to infection, is a major health problem in intensive care unit (ICU) patients worldwide and is associated with high mortality rates [1,2]. Between 500 and 1000 cases of sepsis occur in North America per 100,000 people. The incidence rate in Europe is estimated to range from 400 to 800 cases per 100,000 people. Sepsis mortality varies from 15% to 25% in high-income countries. In its most severe form, sepsis causes profound circulatory and metabolic abnormalities, leading to septic shock, with hospital mortality rates ranging from 30% to 40% [1]. Despite substantial advances in understanding sepsis in recent years, early identification and timely management continue to pose major clinical challenges in intensive care.

Circulating endotoxins contribute to a dysregulated immune response, and extracorporeal removal of endotoxins and cytokines has been suggested as a potential therapy to restore immune balance [3]. Despite a theoretical justification, the current evidence on clinical efficacy and patient outcomes of extracorporeal blood purification remains inconclusive [4,5,6]. Numerous randomized controlled trials and retrospective observational studies have evaluated the effectiveness of endotoxin and cytokine removal across various patient populations, leading to conflicting results. These studies included diverse groups, such as patients with sepsis, severe sepsis, or septic shock, with or without acute kidney injury (AKI), and with or without an indication for renal replacement therapy (RRT).

The effect of cytokine removal in critically ill patients with septic shock complicated by AKI requiring RRT has been evaluated in only four randomized controlled (RCT) small-scale studies, all conducted in China [7,8,9,10]. All these studies demonstrated a positive effect of cytokine removal on hemodynamic parameters, with two of them also reporting lower mortality in the haemoperfusion group. Several European observational studies assessed the effect of cytokine removal in patients meeting the same inclusion criteria [11,12,13]. Schittek et al. found that the implementation of haemoadsorption in patients with septic shock and AKI did not result in lower mortality, shorter ICU stay, reduced catecholamine use, or shorter RRT duration [11]. In contrast, Brouwer et al. reported that cytokine removal was associated with decreased all-cause mortality at 28 days compared to RRT alone [12]. Garcia et al. found an increased risk of mortality in patients treated with haemoadsorption [13]. Rugg et al. demonstrated significantly lower mortality among patients with septic shock receiving CRRT when CytoSorb^®^ was added [14].

In this study, we aimed to evaluate whether combining haemoadsorption therapy with continuous RRT (CRRT) reduces short term (ICU, 30-day, and 60-day) mortality in patients with severe septic shock and AKI requiring RRT.

## 2. Results

**Figure 1:**
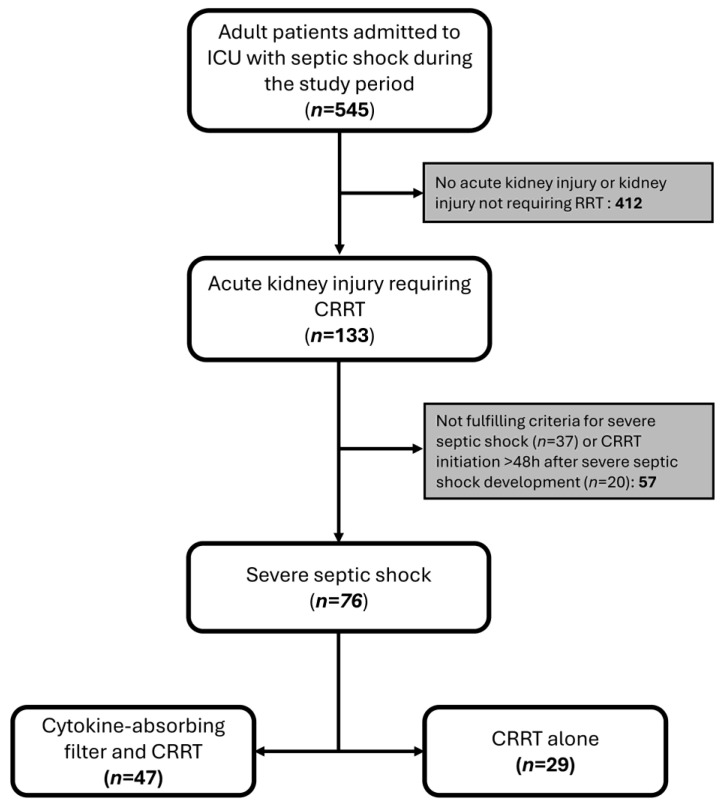
Study flow chart. ICU, intensive care unit; CRRT, continuous renal replacement therapy.

During the study period, a total of 545 patients with septic shock were treated in our ICU. Of these, 133 patients developed AKI requiring CRRT. We identified 76 patients who met the inclusion criteria and included them in the final analysis. Of these, 47 patients were treated with a cytokine-absorbing filter (35 [74.5%] with Oxiris^®^ and 12 [25.5%] with CytoSorb^®^), while 29 patients received CRRT alone (Figure 1).

The demographic and clinical characteristics of the study cohort are described in Table 1. In both groups, most patients were male (34 [72.3%] in the haemoadsorption group and 21 [72.4%] in the CRRT group, *p* = 0.99). The median age in both groups was 66 years. Patients in the haemoadsorption group had a slightly lower Charlson comorbidity index (5 [IQR 2, 6] vs. 6 [IQR 3, 9], *p* = 0.01) but a higher SOFA score (15 [IQR 13, 17] vs. 13 [IQR 11,14], *p* < 0.001) at the initiation of RRT. All patients included in the cohort were mechanically ventilated at the start of RRT. Intrabdominal infections were more common in the haemoadsorption group (55.3% vs. 27.6%, *p* = 0.02). Therapy was initiated in all patients within the first 24 h of the diagnosis of septic shock. In both groups, no side effects were observed during the first 72 h of RRT, apart from mild cases of hypokalaemia and hypophosphatemia.

**Figure 2:**
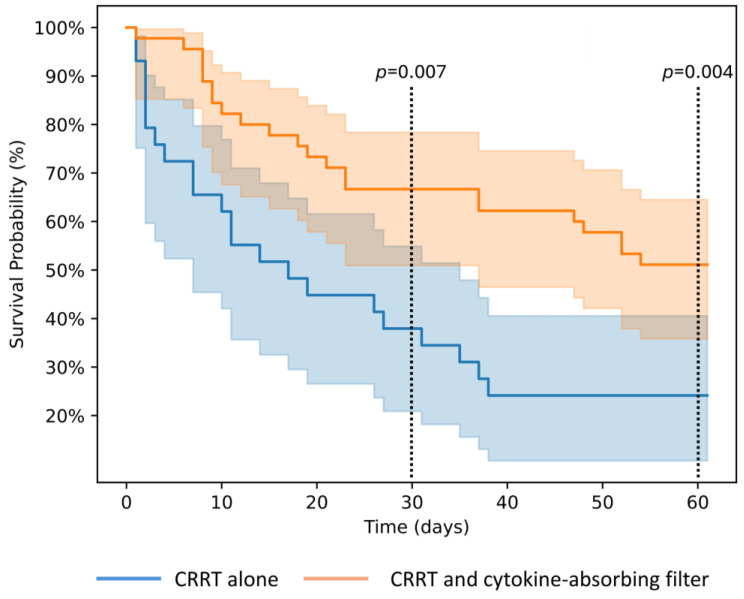
Kaplan–Meier survival curves comparing continuous renal replacement therapy with and without a cytokine-absorbing filter. CRRT, continuous renal replacement therapy.

Blood lactate levels and the VDI were comparable in two groups at the start of RRT. After 24 h of treatment, blood lactate levels did not change significantly in the CRRT alone group (6.4 [IQR 2.65, 11.50] vs. 7.7 [IQR 2.08, 10.25], *p* = 0.33), while the VDI increased slightly (0.41 [IQR 0.24, 0.54] vs. 0.43 [IQR 0.11, 1.05], *p* = 0.009). In contrast, the haemoadsorption group showed a significant reduction in blood lactate levels (5.1 [IQR 3.43, 7.68] vs. 2.3 [IQR 1.6, 3.48], *p* < 0.001) and VDI (0.42 [IQR 0.28, 0.56] vs. 0.15 [IQR 0.05, 0.28], *p* < 0.001). The degree of change in lactate levels and VDI was significantly greater in the haemoadsorption group. Patients treated with a combination of haemoadsorption therapy and CRRT had significantly lower ICU, 30-day, and 60-day mortality rates compared to those receiving CRRT alone (34.0% vs. 65.5%, *p* = 0.008, 34.0% vs. 62.1%, *p* = 0.02, and 48.9% vs. 75.9%, *p* = 0.02 respectively). Kaplan–Meier survival curves are presented in Figure 2. In both groups, the leading cause of death was multiorgan failure, reported in 17 of 23 patients (73.9%) in the haemoadsorption group and 19 of 22 patients (86.4%) in the CRRT group (*p* = 0.30). Brain death was declared in four patients (18.2%) who died in the haemoadsorption group and in three patients (13.6%) in the CRRT group.

After incorporating a multivariate regression model that accounted for age, gender, Charlson Comorbidity Index, SOFA score, VDI, lactate levels at the initiation of treatment, and year of admission, haemoadsorption therapy was independently associated with lower ICU mortality rates (OR 0.16, 95% CI 0.05, 0.50, *p* = 0.002). The only other factor independently associated with ICU mortality was the SOFA score (OR 1.19, 95% CI 1.01, 1.41, *p* = 0.05). The c-index of the model was 0.71 (95% CI 0.59, 0.81).

Logistic regression revealed a significant association between haemoadsorption therapy and 30-day survival (OR 0.13, 95% CI 0.04, 0.48, *p* = 0.002). Other factors independently associated with higher 30-day mortality were male gender (OR 4.64, 95% CI 1.23, 17.45, *p* = 0.02) and SOFA score (OR 1.25, 95% CI 1.03, 1.52, *p* = 0.03). The c-index of the model was 0.78 (95% CI 0.68, 0.87).

However, haemoadsorption therapy was not associated with higher survival after 60 days. Factors independently associated with 60-day mortality were age (OR 1.05. 95% CI 1.01, 1.10, *p* = 0.02) and Charlson Comorbidity Index (OR 1.51, 95% CI 1.19, 1.92, *p* < 0.001). The c-index of the model was 0.75 (95% CI 0.64, 0.84).

## 3. Discussion

In this single-centre retrospective cohort study of patients with severe septic shock and AKI requiring RRT, the addition of a cytokine removal filter to CRRT was associated with improved lactate clearance, faster reduction in VDI, and lower ICU and 30-day mortality rates. The association with 60-day mortality was no longer statistically significant after adjusting for potential cofactors. The survival curves indicate that the differences between the groups are most pronounced during the initial days following the start of therapy while the 60-day mortality is mainly determined by age and comorbidities.

The pathophysiology of sepsis is highly complex and influenced by a multitude of factors, including proinflammatory and anti-inflammatory cytokines, bacterial exotoxins released by injured cells, and host-specific factors such as activated complement and procalcitonin. Inflammation in sepsis can result in severe immune system dysfunction, ranging from a destructive maladaptive systemic inflammatory response syndrome to profound immunosuppression, potentially progressing to multisystem organ dysfunction and death. The physiological rationale for extracorporeal cytokine removal is to restore balance between pro-inflammatory and anti-inflammatory mediators. Elevated circulating levels of cytokines such as TNF-α, IL-1β, IL-6, IL-8, and IL-10 have been linked to increased morbidity and mortality in patients with sepsis, suggesting that their removal could be beneficial for treatment [4]. In addition to cytokines, bile acids, myoglobin, and free haemoglobin are also removed, which may be beneficial in addressing liver and kidney injury, as well as supporting vascular tone and microvascular density [15,16].

Cytokine adsorption, using both Oxiris^®^ and CytoSorb^®^, was evaluated in multiple studies addressing various populations of patients and producing controversial results and a level of evidence that is often considered insufficient to recommend the use of these techniques in routine medical practice [5,6,17,18,19]. Selecting patients with a high likelihood of benefiting from haemoadsorption therapy, as well as determining the optimal timing for therapy initiation, remains a significant challenge [19]. Previous studies have suggested that the intervention is more likely to benefit patients with refractory shock, characterized by high doses of vasopressor support and early multiple organ failure [19,20,21]. Once the appropriate patient population is identified, the endpoints of clinical trials should be clearly defined, encompassing both pathophysiological and clinical outcomes. A reduction in vasopressor requirements, for instance, represents a meaningful clinical outcome that may lead to additional benefits [19,22]. It is crucial to avoid dismissing a treatment as “ineffective” solely because it does not impact a solid endpoint such as survival [22]. The lack of association between the type of filter used during the first 72 h of admission and both intermediate- and long-term mortality is not surprising, as recovery from severe insults such as septic shock depends on multiple factors, including disease severity, treatment effectiveness, and the patient’s psychological resilience, as indicated by age and comorbidities.

In the current study, we included patients with severe septic shock, defined as those requiring high doses of vasopressor agents to maintain adequate organ perfusion, along with end-organ damage characterized by respiratory failure necessitating mechanical ventilation and AKI requiring RRT. The ICU, 30-day, and 60-day mortality rates in this high-risk group of patients were 46.1%, 44.7%, and 59.2%, respectively. The mortality rates in our cohort are consistent with previous reports on patients with septic shock requiring high doses of vasopressors [23]. Additionally, the observed ICU mortality rates are comparable to, and even lower than, those predicted by SOFA score [24].

Evidence regarding the efficacy of cytokine adsorption in this high-risk patient group remains limited. A retrospective single-centre study by Brouwer et al. included patients with septic shock treated with CRRT, with and without the CytoSorb^®^ filter [12]. In their cohort, the median SOFA score was 12, compared to 14 in our cohort. The authors reported significantly lower 28-day mortality rates among patients receiving haemoadsorption therapy. Schittek et al. conducted a single-centre retrospective study involving patients with septic shock treated either with CRRT alone or CRRT combined with a CytoSorb^®^ filter [11]. An extremely high ICU mortality rate of 70% was reported in their cohort. The authors found no significant effect of haemoadsorption therapy on mortality or ICU length of stay. However, the use of haemoadsorption was associated with a reduced duration of RRT, mechanical ventilation, and catecholamine dependency. These findings are consistent with existing RCTs, which also found no reduction in mortality associated with cytokine adsorption [21,25]. Garcia et al. evaluated the effect of cytokine adsorption using the CytoSorb^®^ filter in a cohort of 48 patients with severe, refractory septic shock, IL-6 levels ≥ 1000 ng/L, and an indication for RRT [13]. The included patients had a mean SOFA score of 14 and profound lactatemia (mean blood lactate level of 5.8 mmol/L), parameters comparable to those in our cohort. After matching with a control cohort, haemoadsorption treatment was not associated with reduced IL-6 levels or vasopressor requirements and was linked to an increased hazard of death. Rugg et al. conducted another retrospective single-centre study evaluating patients treated with CytoSorb and renal replacement therapy for septic shock, comparing them to a matched cohort receiving RRT without CytoSorb [14]. Both in-hospital and 28-day-mortality were significantly lower in the haemoadsorption group.

Our study included a highly selected group of patients with septic shock requiring high doses of vasopressors and significant end-organ damage necessitating RRT and mechanical ventilation. As previously suggested, patients with persistent hyperdynamic shock and reduced vascular responsiveness to catecholamines may have a higher likelihood of benefiting from cytokine removal therapy [19]. Although we adjusted our findings for various potential confounders, fully accounting for the complex interactions between the disease, outcomes, and concurrent medical interventions remains a significant challenge.

The current study has some limitations. First, it was a single-centre observational study with a limited sample size and limited follow-up period. Second, the decision to perform CRRT with haemoadsorption rather than CRRT alone was entirely at the discretion of the attending physician, potentially introducing selection bias. Third, two different cytokine-absorbing filters (Oxiris^®^ and CytoSorb^®^) were used during the study period. These membranes may have distinct characteristics and efficacy parameters. Malard et al. examined the effects of in vitro hemoperfusion with the Oxiris^®^ and CytoSorb^®^ hemoperfusion devices. Heparinized human plasma was pre-incubated with pathological quantities of cytokines and filtered in a closed-loop circulation model for two hours, and the removal of 27 cytokines for each device was examined. The removal rates of cytokines and other inflammatory mediators were similar between these devices. However, there was minimal endotoxin removal with CytoSorb^®^ compared to Oxiris^®^ [26,27]. Both filters have been evaluated in numerous studies examining their effects in various critically ill populations [4,5]. Fourth, while patients were generally discharged from the ICU after achieving clinical stability, individual discharge decisions varied, which may have contributed to differing ICU mortality rates over time. Fifth, our database does not include laboratory parameters such as CRP, procalcitonin, IL-6, and TNF-α levels.

## 4. Materials and Methods

- Study design and data sources

We conducted a single-centre retrospective cohort study using data from patients hospitalized in an 18-bed general ICU at Rambam Health Care Campus (RHCC) between 1 January 2018, and 28 February 2024. RHCC, located in Haifa, Israel, is a 1000-bed tertiary academic hospital serving over two million residents in northern Israel. According to hospital records, there are 80,000 to 90,000 inpatient admissions annually.

The study was approved by the Institutional Review Board at RHCC (approval number RMB-D-0326-24). The need for written informed consent was waived due to the retrospective study design. Data were analysed anonymously.

Data analysed in this study were retrieved from Prometheus, the RHCC integrated electronic medical records system, and from the ICU patient data management system Metavision (iMDsoft, Tel Aviv, Israel).

The Strengthening the Reporting of Observational Studies in Epidemiology (STROBE) guideline was used to ensure proper reporting of methods, results, and discussion [28].

- Participants and RRT protocol

Adult patients (≥18 years old) admitted with severe septic shock or those who developed severe septic shock during their ICU stay and required CRRT were deemed eligible for this study.

Exclusion criteria included: (1) presence or suspicion of cardiogenic shock, haemorrhagic shock, obstructive shock, or anaphylactic shock; and (2) initiation of blood purification or CRRT treatment more than 48 h after the diagnosis of septic shock.

Septic shock was defined according to the Third International Consensus Definitions for Sepsis and Septic Shock (Sepsis-3) [2]. Briefly, these patients exhibited life-threatening organ dysfunction (indicated by an acute increase in the Sequential Organ Failure Assessment (SOFA) Score of ≥2 points due to infection) and required vasopressors to maintain a mean arterial pressure (MAP) of ≥65 mmHg, along with a serum lactate level > 2 mmol/L (18 mg/dL) despite adequate volume resuscitation. For this study, we defined severe septic shock as shock requiring the administration of at least two vasopressors and corticosteroids to maintain the MAP target. During the study period, norepinephrine was used as the first-line vasopressor for patients with septic shock. Vasopressin and corticosteroids (intravenous hydrocortisone at a dose of 300 mg/day, given as 100 mg every eight hours) were typically initiated when the norepinephrine dose required to maintain MAP ≥ 65 mmHg exceeded 0.25 mcg/kg/min [29]. CRRT was initiated in accordance with the Kidney Disease: Improving Global Outcomes (KDIGO) Clinical Practice Guidelines for Acute Kidney Injury [30].

All patients were treated with continuous venovenous hemodiafiltration using a hemofilter, either through the PrisMax CRRT set (Baxter, Deerfield, IL, USA) or the Aquarius platform (Nikkiso Europe GmbH, Hannover, Germany). Blood flow rates were maintained between 150 and 200 mL/min, with a therapeutic dosage (i.e., the sum of dialysate and ultrafiltrate flow rates) of 30–35 mL/kg/h. Replacement fluid was delivered into the extracorporeal circuit both before and after the filter (i.e., predilution and postdilution), with a typical dialysate-to-replacement fluid ratio of approximately 1:1:1. Bicarbonate-buffered solutions (Prism0CAL, Hemosol B0, or Phoxilium, Baxter, Deerfield, IL, USA) were used as the dialysate and replacement fluids. Anticoagulation was managed using unfractionated heparin or regional citrate anticoagulation. All the filters were changed after 48–72 h of use.

Given the lack of clear recommendations regarding specific indications for haemoadsorption therapy in septic shock, no protocol was implemented during the study period for allocating patients to the CRRT and haemoadsorption group; the decision was made at the discretion of the treating intensive care physician.

In the CRRT-alone group, RRT was performed using the AQUAMAX^®^ HF12 (Nikkiso Europe GmbH, Hannover, Germany) or the Prismaflex M150 Set (Baxter, Deerfield, IL, USA) as long as an indication for RRT existed.

In the haemoadsorption group, therapy was conducted using either CytoSorb^®^ (CytoSorbents Corporation, Monmouth Junction, NJ, USA) or Oxiris^®^ (Baxter, Deerfield, IL, USA). After completing 72 h of treatment with a cytokine-adsorbing filter, a standard hemofilter (AQUAMAX^®^ HF12 or Prismaflex M150 Set) was used as a replacement, and treatment was continued as long as an indication for RRT existed. In cases of premature clotting (<72 h from initiation), the filter was replaced with another cytokine-absorbing device.

In both groups, CRRT was discontinued if there was an improvement in renal function. Patients were treated according to local protocols as part of standard care, with no additional interventions applied specifically for the purpose of this study. Data were collected retrospectively.

- Data collection and endpoints

The data collected included demographics, Charlson Comorbidity Index at admission [31], infection source, SOFA score at CRRT initiation [24], arterial lactate level at CRRT initiation and after 24 h, vasopressor dependency index (VDI) at CRRT initiation and after 24 h, and ICU and in-hospital all-cause mortality. Data collection was carried out by well-trained research staff members to ensure minimal bias. The VDI was calculated using the following formula: [(dopamine dose × 1) + (dobutamine dose × 1) + (adrenaline dose × 100) + (noradrenaline dose × 100) + (vasopressin × 100)]/MAP. Epinephrine, norepinephrine, dobutamine, and dopamine are expressed as µg/kg/min and vasopressin as units/min [32].

The primary endpoint was ICU mortality, comparing haemoadsorption therapy to CRRT alone. Secondary endpoints included 30-day and 60-day all-cause mortality, differences in VDI at CRRT initiation and after 24 h of therapy, and differences in lactate levels at CRRT initiation and after 24 h of therapy between the haemoadsorption group and the control group.

- Statistical analysis

Patients’ characteristics were summarized using descriptive statistics. Categorical variables were presented as frequencies and percentages, while continuous variables were reported as medians with interquartile ranges (IQR). Differences between categorical variables were analysed using the χ^2^ test, and the Mann–Whitney U and Wilcoxon tests were employed to compare continuous variables. Unadjusted 30 and 60-day survival analysis was performed using the Kaplan–Meier curves. Forward logistic regression modelling was used to analyze the relationship between short-term mortality and haemoadsorption, adjusting for potential confounders, including age, gender, Charlson Comorbidity Index, SOFA score, VDI, and lactate level. Given the extended study period, we also examined the effect of time trends (i.e., year of admission) on the analysis results. Associations between potential risk factors and mortality were quantified using odds ratios (OR) and 95% confidence intervals (95% CI). The models’ discriminative ability was evaluated using a concordance index (c-index). A *p*-value of ≤0.05 was considered statistically significant. All available data from our databases within the study time frame were utilized. Missing data were addressed using listwise deletion. Data analysis was performed using SPSS version 25.0 (IBM Corp, Armonk, NY, USA) and MedCalc for Windows, version 15.0 (MedCalc Software, Ostend, Belgium).

## 5. Conclusions

In conclusion, cytokine adsorption in severe septic shock complicated by AKI requiring CRRT is associated with reduced vasopressor requirements, improved blood lactate clearance, and better short-term mortality outcomes. Further high-quality RCTs are needed to assess the effects of haemoadsorption in critically ill patients with severe septic shock and AKI.

## Figures and Tables

**Table 1:** Demographic and clinical characteristics of 76 patients included in the study.

	Cytokine-Absorbing Filter and CRRT (*n* = 47)	CRRT Alone (*n* = 29)	*p*-Value
Male gender, *n* (%)	34 (72.3%)	21 (72.4%)	0.99
Age, years, median (IQR)	66 (51.0, 70.8)	66 (55.3, 74.0)	0.38
Charlson comorbidity index, median (IQR)	5 (2, 6)	6 (3, 9)	***0.01***
Infection source, *n* (%)			
Pulmonary	3 (6.4%)	6 (20.7%)	0.10
Abdominal	26 (55.3%)	8 (27.6%)	
Urogenital	5 (10.6%)	4 (13.8%)	
Skin, muscle, bone infection	6 (12.8%)	7 (24.1%)	
Other	7 (14.9%)	4 (13.8%)	
Sequential Organ Failure Assessment Score at initiation of CRRT, median (IQR)	15 (13, 17)	13 (11, 14)	***<0.001***
Lactate, mmol/L, median (IQR)			
Before treatment	5.1 (3.43, 7.68)	6.4 (2.65, 11.50)	0.28
After 24 h	2.3 (1.6, 3.48)	7.7 (2.08, 10.25)	***<0.001***
∆ Lactate	−2.10 (−4.23, −0.90)	−0.50 (−2.75, 1.2)	***0.02***
Vasopressor Dependency Index, median (IQR)			
Before treatment	0.42 (0.28, 0.56)	0.41 (0.24, 0.54)	0.64
After 24 h	0.15 (0.05, 0.28)	0.43 (0.11, 1.05)	***0.002***
∆ Vasopressor Dependency Index	−0.23 (−0.38, −0.11)	0.00 (−0.25, 0.44)	***<0.001***
Weaning from mechanical ventilation, *n* (%)	23 (48.9%)	8 (27.6%)	0.07
ICU mortality, *n* (%)	16 (34.0%)	19 (65.5%)	***0.008***
30-day all-cause mortality, *n* (%)	16 (34.0%)	18 (62.1%)	***0.02***
60-day all-cause mortality, *n* (%)	23 (48.9%)	22 (75.9%)	***0.02***

CRRT, continuous renal replacement therapy; IQR, interquartile range; ICU, intensive care unit. Statistically significant values (*p* ≤ 0.05) are given in bold and italics.

## Data Availability

The data that support the findings of this study are available from the corresponding author, upon reasonable request.
